# Nurses and Trade Unions

**Published:** 1921-02-12

**Authors:** 


### THE EDITOR'S LETTER-BOX.

**Correspondence on all subjects is invited, but we cannot in any way be responsible for the opinions expressed by our correspondents, who must give their name and address as a guarantee of good faith, but not necessarily for publication. Correspondents are reminded that brevity of style and conciseness of statement greatly facilitate early insertion.**

### Nurses and Trade Unions.

*To the Editor of* THE HOSPITAL.

Sir,—In a recent *Sunday Chronicle* appears a rousing article on financial affairs by Sir Ernest E. Wild, K.C., M.P., and referring to the middle classes, who have, in his opinion, "a dreadful time under every Government," Sir Ernest says that "it is largely their own fault. Nobody ever thinks of them because they are not highly organised in trade unions."

I presume I am right in thinking that nurses belong to the middle class, and also that nobody ever thinks of them. At any rate, our outstanding grievances are taking an intolerably long time to remedy, and I am beginning to wonder if Sir Ernest Wild is not right, and that the reason is because we are not highly organised in trade unions.

Now, Sir, our leaders, including the nursing press, deprecate nurses joining a trade union, and, speaking for myself, I have never been favourable to the idea. But our leaders cannot have it every way. If we hold aloof from trade unionism we expect our leaders to act as belligerents in our behalf. I have heard this subject warmly discussed, and I think it would help to clear the air if some of the College authorities would make clear exactly how we stand and what we are to expect. Is it not true that Lord Knutsford says that the College can do nothing? Sir Ernest Wild does not sympathise with the middle classes in their plight. He says "it is largely their own fault." Shall we receive the same consolation one of these days?

The College elections are coming along, and I see well-known names amongst the nominees. But there is no indication of their policy or intentions, if elected. Are we not entitled to know? What we need particularly at the present moment is an assurance direct from the College Council that a forward policy will be pursued with vigour, and that our Union, if it is not a trade union, will not be weaker than one, but stronger and more influential.

If, as Sir Ernest Wild suggests, the Union is the thing, then it seems to me that we ought to avail of it. It is quite clear that if we back the wrong horse we shall be told when it is too late that it is our own fault.

Sister Caroline.

